# Editorial: Nanoparticles for cancer immunotherapy: from basics to clinics

**DOI:** 10.3389/fimmu.2025.1668580

**Published:** 2025-08-12

**Authors:** Fernando Torres Andón, Paola Allavena, María José Oliveira, Clement Anfray

**Affiliations:** ^1^ Medical Oncology Department, Fundación Pública Galega de Investigación Biomédica (INIBIC), Complexo Hospitalario Universitario de A Coruña (CHUAC), A Coruña, Spain; ^2^ Clinical and Research Hospital Humanitas, Istituto di Ricovero e Cura a Carattere Scientifico (IRCCS), Milan, Italy; ^3^ Tumour and Microenvironment Interactions Group, i3S-Instituto de Investigação e Inovação em Saúde da Universidade de Porto, Porto, Portugal; ^4^ Université de Caen Normandie, CNRS, Normandie Université, ISTCT UMR6030, GIP CYCERON, Caen, France

**Keywords:** nanoparticles, cancer immunotherapy, tumor microenvironment, drug delivery, combination therapy, cancer resistance, theragnostic nanomedicine

Antitumoral immunotherapies have achieved remarkable results in the treatment of a variety of cancers, but there are also limitations such as low response or side effects. An effective immune response against cancer requires the appropriate activity and interaction of cellular and molecular mediators involved in the “cancer-immunity cycle” to prevent tumor progression, fight against cancer cells and ultimately achieve tumor eradication (1). Nanoparticles (NPs) have been engineered to improve the delivery of a wide range of immunotherapies (including small drugs, nucleic acids, peptides, or antibodies) after intravenous administration towards tumor sites, but also for other routes of administration with the aim to improve their efficacy and/or safety (2). Nanotechnological approaches have been developed and evaluated to: i) dismantle the immunosuppressive signals in the tumor microenvironment (TME), ii) activate specific molecular or cellular mediators in the TME, iii) improve the transport of antigens toward the appropriate immune cells, in the context of cancer vaccines, iv) induce immunogenic cell death, thus killing cancer cells and activating antitumor immune responses, and lately v) improve cell therapies or vi) advance the diagnosis of cancer progression through the tracking or quantification of molecular and cellular immune mediators. In the next years, new research will have the potential to provide exciting results improving tissular and cellular targeting but also applying stimuli-responsive NPs (i.e. pH, temperature, ultrasounds or radiotherapy), drug delivery strategies for combination therapies (i.e. chemo-immunotherapy or immuno-radiosensitizers) or other innovative approaches intended to overcome cancer resistance (3).

The TME of solid tumors is characterized by a state of immunosuppression, mainly caused by myeloid cells and Treg lymphocytes (4). Anfray et al. have used polymeric nanocapsules loaded with agonists of the Toll-like receptors (TLR) to activate the immune component of tumor tissues. As published before (5), they found that the combination of two TLR ligands: poly(I:C) and resiquimod, is much more effective in triggering an anti-tumor immune response than the single monotherapies. Poly(I:C) plus resiquimod, encapsulated in protamine nanocapsules (NC) were intratumorally injected and strongly inhibited tumor growth in different murine preclinical models. These TLR-loaded-protamine-NCs were also coated with an additional layer of hyaluronic acid polymer functionalized with mannose to improve the targeting of tumor-associated macrophages (TAMs) upon intravenous administration, showing similar antitumor efficacy and no systemic toxicity. Mechanistically, these NCs triggered local antitumor immunity by reprogramming TAMs into M1-like macrophages cytotoxic towards tumor cells.

Another strategy to reactivate tumor-infiltrating leukocytes is to use immunostimulatory cytokines. As the direct injection of cytokines is limited by their short half-life in the circulation, Li et al. used a cocktail of mRNA coding for IL-12, IL-7 and IFN-α. These three cytokines are key mediators of the Th1 immune response and enhance the activation and cytotoxicity of CD8^+^ T and NK cells. The mRNA cocktail was encapsulated in lipid nanoparticles (LNP) and administered intratumorally. In multiple syngeneic mouse models, the treatment induced tumor regression and antitumor immune memory. Mechanistically, cytokine mRNAs induced inflammation of the TME, characterized by robust T cell infiltration and significant inflammatory cytokine and chemokine production. Additionally, this mRNA cocktail further enhanced the antitumor efficacy of checkpoint blockade immunotherapy.

In a review article Zhang et al. highlighted the advantages of nanodrug delivery systems (NDDS) to improve cancer immunotherapies. NDDS carrying specific antitumor compounds have high stability and biocompatibility, improving the solubility of hydrophobic drugs, prolonging the drug cycle, enhancing permeability through physiological barriers, better targeting delivery at tumor sites and reduced toxic side effects. The custom design of NDDS with specific ligands or antibodies for cell receptors, but also nucleic acids, proteins and polysaccharide molecules, has now reached a high level of complexity, to better target cancer cells, immune cells in the TME and also specific metabolic conditions. A similar focus on NP systems targeting the TME, but with a particular focus on head and neck squamous cell carcinomas (HNSCCs) was provided by Zhang et al. They focused on the non-cellular components of the TME: the blood network and the extra-cellular matrix (ECM). Blood vessel normalization is an important goal for enhancing drug delivery and reducing tumor growth, and neo-angiogenesis is a driving force of malignant transition and aggressiveness. NPs loaded with angiogenesis inhibitors showed interesting results and are being evaluated in clinical studies. Degradation of the ECM scaffold is an essential hallmark of progressing tumors, operated by matrix metalloproteinases (MMPs) and other enzymes. NPs responsive to MMPs have been developed and exhibited great potential for the treatment of solid tumors.

Another difficult-to-treat tumor is ovarian cancer: initially responding to current chemotherapy, ovarian cancer resistant cells frequently arise later on and cause disease relapse. The review by Li et al. reports the efforts of delivering natural drugs to treat ovarian cancer using nanoscale delivery systems. Potentially active natural drugs against ovarian tumors include flavonoids, polysaccharides, alkaloids, quinones and others. Several nanotechnological approaches have been used to improve the delivery of these natural compounds, control their release, extend their half-life in circulation and maximizing their therapeutic activity. Another contribution on ovarian cancer by Xiong et al. focused on the role of nanotechnology in delivering chemo-immunotherapy to target the immune and non-immune TME. Particular attention was devoted to NPs with ability to activate immune effector cells or to block immunosuppressive cells, but also to NPs targeting cancer cells, inducing their apoptosis or intervening in the mechanisms of EMT occurrence.

Another feature of the immunosuppressed TME is the paucity of effector T cells. The adoptive transfer of tumor-specific CAR-T cells in solid tumors is frequently hampered by their poor tissue trafficking ability. To solve this challenge Pfister et al. developed superparamagnetic iron oxide nanoparticles (SPIONs) that can make cells magnetically controllable for site-specific enrichment. The magnetic guidability of SPIONs has been previously used to enrich therapeutic drugs in a desired region (6), however, it was unclear if SPION-loading had an impact on T cell function. Now, the authors demonstrated that SPION-loading of human T cells did not affect cellular activity or the functionality of the T cell receptor (TCR). SPION-loaded T cells retained their ability to proliferate, express activation markers, produce cytokines and effectively killed tumor cells. In another article, Yang et al., developed iron NPs (PEG-Fe3O4) to treat breast cancer. In addition to drug delivery carriers, iron NPs could serve as an iron source, massively increasing the cellular levels of irons, leading cancer cells to ferroptosis. However, complement activation via the C5a/C5aR pathway can drive resistance to ferroptosis. Therefore, the authors loaded the iron NPs with a C5a receptor antagonist. The novel PEG-Fe_3_O_4_@C5aRA induced more powerful cancer cell ferroptosis than PEG-Fe_3_O_4_, and complement inhibition by these NPs restrained M2-polarization of macrophages, showing significant therapeutic efficacy. Differently, Li et al. presented a facile method to develop personalized cancer vaccines by embedding tumor antigens within iron-based metal organic frameworks through the interaction between Fe^3+^ ions and endogenous fumarate ligands. These iron-based NPs allowed high tumor antigen loading coupled with adjuvant activity. By adjusting the synthesis parameters, the morphology of these cancer vaccines can be tailored from microscale to nanoscale. The smaller format showed higher antitumoral activity in lung cancer models, presumable due to enhanced lymph node targeting.

Nanoparticle-based hyperthermia has been used to promote the selective death of tumors by raising cell temperature. Zhang et al. reviewed currently available hyperthermia modalities, including near-infrared laser or alternating magnetic field with ability to cause cell death via apoptosis or necrosis, respectively. Hyperthermia not only kills tumor cells directly, but also activates the immune system via different mechanisms (i.e. immunogenic cell death), and combinations with additional immunotherapies to boost effective antitumor immune responses are under investigation.

Exosomes and extracellular vesicles (EVs) are small particles secreted by numerous cell types that circulate in almost all body fluids, acting as crucial messengers for cell-to-cell communication. Due to their nanometer size (30–150 nm and 150–400 nm, respectively) these vesicles can be considered naturally occurring NPs. The review by Li et al. summarizes the role of therapeutic EVs targeting the TME of melanoma tumors. EVs released by immune cells have attracted much attention for cancer immunotherapy, because they display immunological features of their parental cells. For instance, T cell-derived EVs exert anti-tumor effects and EV released by IL2-stimulated CD4+ T lymphocytes increase the cytotoxicity of CD8+T cells. Cecchetti et al. have studied the therapeutic activity of NK cells-derived extracellular vesicles (NKEV) towards hematopoietic tumors. NKEV isolated from *in vitro* amplified healthy human NK cells demonstrated a direct cytotoxic effect against human B cell lymphoma, significantly reducing tumor growth in preclinical *in vivo* models.

Overall, NP-based therapies have emerged as a revolutionary approach to cancer treatment by offering several advantages compared to traditional pharmacological methods. Even if only a few nanomedicines, mainly liposomes and basic NP systems, are - so far - approved for clinical use (Zhang et al.), basic and translational research are extremely active and some ongoing clinical trials are already evaluating the potential of nanotherapy in cancer patients. Novel discoveries in the fields of cancer immunotherapy and nanotechnology fuel hope for the future.
